# Development and validation of early death risk score model for emergency status prediction in very severe aplastic anemia

**DOI:** 10.3389/fimmu.2023.1175048

**Published:** 2023-04-20

**Authors:** Xu Liu, Wenrui Yang, Li Zhang, Liping Jing, Lei Ye, Kang Zhou, Yuan Li, Jianping Li, Huihui Fan, Yang Yang, Youzhen Xiong, Xin Zhao, Fengkui Zhang

**Affiliations:** ^1^ State Key Laboratory of Experimental Hematology, National Clinical Research Center for Blood Diseases, Haihe Laboratory of Cell Ecosystem, Institute of Hematology & Blood Diseases Hospital, Chinese Academy of Medical Sciences & Peking Union Medical College, Tianjin, China; ^2^ Tianjin Institutes of Health Science, Tianjin, China

**Keywords:** very sever aplastic anemia, early death, risk score model, immunosuppressive therapy, hematopoietic stem cell transplantation

## Abstract

This study developed and validated the Early Death Risk Score Model for early identification of emergency patients with very severe aplastic anemia (VSAA). All 377 patients with VSAA receiving first-line immunosuppressive therapy (IST) were categorized into training (n=252) and validation (n=125) cohorts. In the training cohort, age >24 years, absolute neutrophil count ≤0.015×10^9^/L, serum ferritin >900ng/mL and times of fever before IST >1 time were significantly associated with early death. Covariates were assigned scores and categorized as: low (score 0-4), medium (score 5-7) and high (score ≥8) risk. Early death rate was significantly different between risk groups and the validation cohort results were consistent with those of the training cohort. The area under the receiver operating characteristic curve for the model was 0.835 (0.734,0.936) in the training cohort and 0.862 (0.730,0.994) in the validation cohort. The calibration plots showed high agreement, and decision curve analysis showed good benefit in clinical applications. The VSAA Early Death Risk Score Model can help with early identification of emergency VSAA and optimize treatment strategies. Emergency VSAA with high risk is associated with high early death rate, and alternative donor hematopoietic stem cell transplantation could be a better treatment than IST even without HLA-matching.

## 1 Introduction

Idiopathic aplastic anemia (AA) is a disease involving bone marrow failure. It is characterized by bone marrow hypoplasia and pancytopenia, with the main clinical manifestations being infection, hemorrhage, and anemia. Before the 1970s, there was no effective targeted treatment, and the mortality rate of patients with severe aplastic anemia (SAA) was approximately 80%, caused mainly by severe infections and fatal hemorrhagic events (1). Current treatment focuses on early reestablishment and restoration of bone marrow hematopoiesis. For patients younger than 40 years of age with SAA or very severe aplastic anemia (VSAA) with human leukocyte antigen (HLA)-matched sibling donors, guidelines still recommend allogeneic hematopoietic stem cell transplantation (HSCT) to rapidly reestablish bone marrow hematopoiesis (2). For patients not eligible for HSCT, strong immunosuppressive therapy (IST) combined with thrombopoietin receptor agonists (TPO-RAs) is the first-line treatment option to restore partial bone marrow function and reduce the risk of infection and hemorrhage (2). These two effective treatments together reduce the mortality associated with AA and lead to long-term survival in 70-80% patients (3–5).

These strategies may be more appropriate for the SAA cases with relatively high residual hematopoiesis. Whereas patients with VSAA with low residual hematopoiesis and low absolute neutrophil counts (ANC), are at high risk of life-threatening infection and hemorrhage even at the first consultation, and early death may occur in patients treated with standard IST. In addition, studies have shown that response and long-term survival following IST are significantly lower in patients with VSAA than SAA, and such VSAA cases still require salvage therapy (e.g., haploidentical donor HSCT or matched unrelated donor HSCT) (6). Therefore, we believe that these patients should be identified as “emergency” patients at an early stage and treated with HSCT including transplantation from alternative donors as soon as possible, rather than after IST failure. Our study retrospectively analyzed data from patients with VSAA treated with IST with the aim of establishing a predictive early death risk score model to identify “emergency” cases of VSAA and optimize treatment strategies.

## 2 Patients and methods

### 2.1 Patients

Data from 377 consecutive patients were retrospectively analyzed. Patients diagnosed with VSAA and treated with IST at the Anemia Therapeutic Center of the Institute of Hematology & Blood Diseases Hospital, Chinese Academy of Medical Sciences, between July 2010 and December 2019 were included. In accordance with the Declaration of Helsinki, all patients voluntarily provided written informed consent. The study was approved by the appropriate institutional review boards and ethics committees of involved institutions. AA was diagnosed with reference to the International Agranulocytosis and Aplastic Anemia Study Group criteria (1987) (7), and disease severity staging was defined according to the Camitta criteria (1976) (8).

### 2.2 Immunosuppressive therapy and supportive care

All patients were treated with porcine anti-human T lymphocyte immunoglobulin (ATG) and cyclosporine A (CsA) because of the ineligibility for HSCT. None of the patients were treated with TPO-RAs which were unavailable in China before 2019. Porcine ATG was administered intravenously at a dose of 20 mg/kg/d for five consecutive days. The initial dose of CsA was 3 mg/kg/day orally twice daily, then adjusted to achieve a trough concentration of 150-250 ng/mL and peak concentration of 700-1000 ng/mL. CsA was tapered after the patients had achieved an optimal hematologic response for at least three months. Patients were given red blood cell or platelet (PLT) transfusion support therapy until they obtained an optimal hematologic response. Granulocyte colony-stimulating factor (G-CSF) was routinely administered at 5 μg/kg/d to patients with ANC <0.5×10^9^/L and was discontinued once ANC remained >0.5×10^9^/L for more than 7 days.

### 2.3 Hematologic response and follow-up

Complete response (CR) was defined as hemoglobin (HGB) >100 g/L, ANC >1.5×10^9^/L, and PLT >100×10^9^/L. Partial response (PR) was defined as independence from red blood cell and PLT transfusions, improving hematologic parameters and no longer meeting the SAA criteria. No response (NR) was defined as continuous transfusion dependence or the hematological parameters still meting the SAA criteria.

Patient follow-up assessments were completed by April 2022. Overall survival (OS) was defined as the time from IST until death or last follow-up. Event-free survival (EFS) was defined as the time from IST to any of the following events: (1) receiving HSCT or a second round of IST; (2) relapse; (3) clonal evolution to hemolytic PNH, acute myeloid leukemia or myelodysplastic syndrome; (4) persistent NR at 12 months of IST; or (5) death.

### 2.4 Adverse outcomes

Early death was defined as death within 3 months of IST. Response to G-CSF was defined as ANC>0.2×10^9^/L within 7 days after administration of G-CSF. Fever was defined based on established literature (9) as a single oral temperature >38.3 or ≥38°C for at least 1 h. Fever times were defined as the time of fever onset to the time the patient’s temperature had fallen below 37.5°C for 3 consecutive days without non-steroidal anti-inflammatory drug or glucocorticoid treatment. Fever duration was defined as the number of days from the beginning to the end of 1 fever episode. A hemorrhagic event was defined as active systemic intracranial, pulmonary, or gastrointestinal bleeding.

### 2.5 Statistical analysis

Patients were assigned to one of the training and validation cohorts at a 2:1 ratio by stratified randomization, with early death as an outcome variable. Descriptive statistics were calculated for the overall cohort as well as for the training and validation cohorts. Continuous variables are expressed as medians and ranges and compared using Mann–Whitney U tests. Categorical variables are expressed as counts and compared using chi-square tests. The receiver operating characteristic (ROC) curve was used to optimize the cut-off values of each parameter. Logistic regression models were used for univariate and multivariate analyses to recognize variables related to early death. Multi-collinearity among variables was checked by variance inflation factor. Variables with p<0.1 in univariate analyses were included in multivariate analyses. Significant covariates from the training dataset were used to develop the risk score system. Ten-fold cross-validation was used to validate the risk model. Weighted scores were assigned to these covariates according to their regression coefficient (10). The area under the ROC curve (AUC) was used to evaluate the sensitivity and specificity of the model. The calibration curves were plotted using the Hosmer–Lemeshow test. Decision curve analysis was used to evaluate the net benefit of the model. P<0.05 for two-sided tests was considered statistically significant. Analysis was performed using SPSS (version 26.0). Graphs were drawn using GraphPad Prism 8 (La Jolla, CA) and R version 4.2.1 (Vienna, Austria).

## 3 Results

### 3.1 Response and survival profile

**Figure 1:**
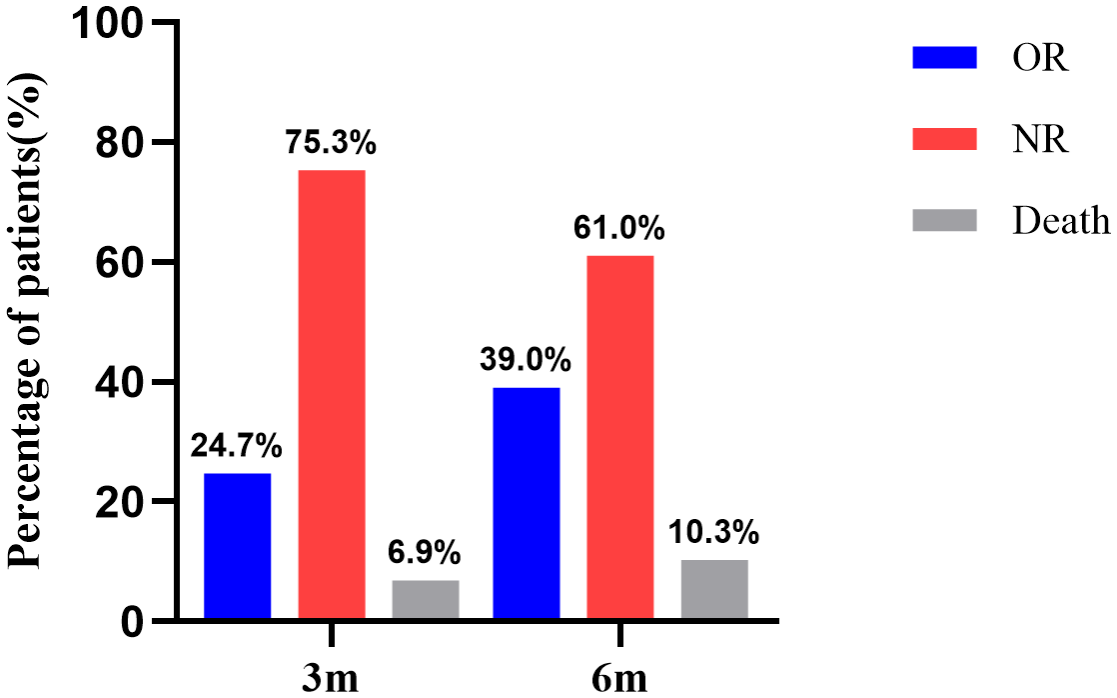
Hematological response rate and death rate at 3 and 6 months.

For all 377 patients, the NR rate was 75.3% (284/377) at 3 months after IST. The hematologic response rate was 24.7% (93/377), including 14 cases of CR and 79 cases of PR. At 6 months after IST, the NR rate was 61.0% (230/377). The hematologic response rate was 39.0% (147/377), including 37 cases of CR and 110 cases of PR (**Figure 1**).

**Figure 2:**
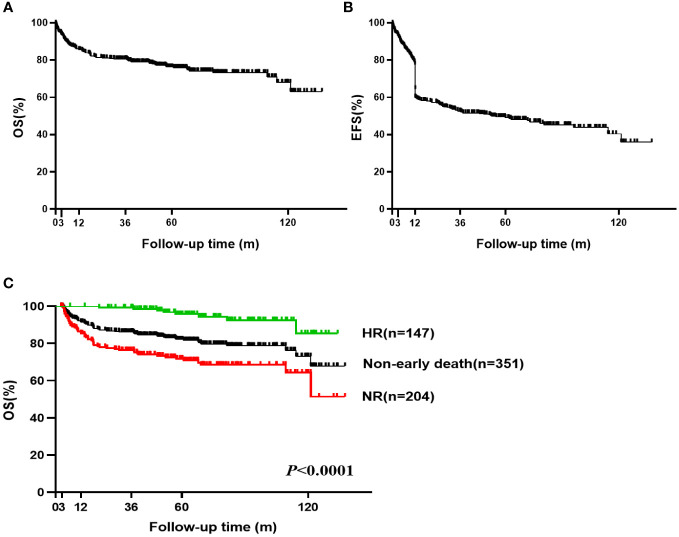
Overall survival (OS) and Event-free survival (EFS) of VSAA. **(A)** The 5-year OS was 76.3% (95% CI, 71.481-80.467) and 10-year OS was 70.0% (95% CI, 58.626-75.648) for all 377 VSAA patients; **(B)** The 5-year EFS was 49.5% (95% CI, 44.192-54.582) and 10-year EFS was 40.4% (95% CI, 31.869-48.769) for all 377 VSAA patients; **(C)** The 5-year OS was 95.8% (95% CI, 90.075-98.238) in non-early death and HR patients (n=147). The 5-year OS was 71.8% (95% CI, 64.671-77.743) in non-early death and NR patients (n=204).

A total of 92 (24.4%, 92/377) patients died, including 26 cases of early death (6.9%, 26/377) and 66 cases of late death. The 5-year OS was 76.3% (95%CI, 71.481-80.467) and 10-year OS was 70.0% (95%CI, 58.626-75.648) (**Figure 2A**). The 5-year EFS rate was 49.5% (95% CI, 44.192-54.582) and the 10-year EFS rate was 40.4% (95% CI, 31.869-48.769) (**Figure 2B**). HSCT was required in 36 patients due to ineffectiveness, relapse or clonal transformation after IST and 29 of these patients achieved CR, three were still recovering, and four died after HSCT.

Among the 351 patients not experiencing early death, the hematologic response rate at 6 months was 41.9% (147/351) (**Supplementary Table 1**). The 5-year and 10-year OS were 82.1% (95% CI, 77.403-85.905) and 73.1% (95% CI, 62.977-80.886), respectively. For non-early death patients who achieved hematologic response after IST, the 5-year OS was 95.8% (95% CI, 90.075-98.238) and 10-year OS was 85.4% (95% CI, 63.118-94.695). Five of these patients (four relapsed and one transformed to myelodysplastic syndrome) were treated with HSCT, and all achieved CR. For patients not experiencing early death but showing NR after IST, the 5-year OS was 71.8% (95% CI, 64.671-77.743) and the 10-year OS was 64.3% (95% CI, 52.653-73.765) (**Figure 2C**). Thirty-one of these patients were treated with subsequent HSCT (27 patients achieved CR and 4 patients died after HSCT).

### 3.2 Emergency VSAA: Early death

All 26 VSAA patients with early death had extremely low median baseline hematological parameters, with ANC 0.005 (0-0.14) ×10^9^/L and PLT 5.5 (0-20) ×10^9^/L. They developed fever during the treatment period, with a median fever duration of 17.5 (1-69) days, and 21 patients had 2 or more occurrences of fever. Twenty-two patients developed fever before IST, with a median fever duration of 5 (0-38) days and 10 patients had 2 or more times of fever before IST. Twenty-five patients with early death had no response to G-CSF therapy. Hemorrhagic events were observed in 42.3% (11/26) patients with early death, and 2 hemorrhagic events occurred before IST. Compared with non-early death patients, VSAA patients with early death had lower baseline hematologic parameters, higher fever rates, longer median fever duration, and higher rates of hemorrhagic events (**Table 1**).

**Table 1:** Features of early death and non-early death patients in VSAA.

Features	Early death (n=26)	Non-early death (n=351)	*P* value
**Median age at diagnosis, years (range)**	38(5-70)	26(6-75)	0.003
**Gender, male/female**	15/11	188/163	0.683
**History, months (range)**	1(0.25-72)	0.75(0.1-132)	0.320
**ANC [×10^9^/L, M (range)]**	0.005(0-0.14)	0.05(0-0.19)	0.000
**HGB [g/L, M (range)]**	57.5(48-72)	60(25-123)	0.534
**PLT [×10^9^/L, M (range)]**	5.5(0-20)	6(0-27)	0.109
**ARC [×10^9^/L, M (range)]**	2.02(0-4.6)	3.5(0.2-38.9)	0.000
**SF [ng/mL, M (range)]**	971.55(105.5-3193)	502.5(48.56-3459)	0.006
**Fever before IST (Yes/No)**	22/4	202/149	0.007
**Fever times before IST, times**	1(0-3)	1(0-3)	0.001
**Median fever duration before IST, days (range)**	5(0-38)	1(0-30)	0.000
**No response to G-CSF (Yes/No)**	25/1	247/104	0.005
**Hemorrhagic events before IST (Yes/No)**	2/24	5/346	0.126

ANC, Absolute neutrophil count; HGB, Hemoglobin; PLT, Platelet; ARC, Absolute reticulocyte count; SF, Serum Ferritin; IST, Immunosuppressive therapy.

### 3.3 Study cohorts

A total of 377 VSAA patients were assigned to training (n=252) and validation (n=125) cohorts by stratified randomization. Clinical characteristics in the training and validation cohorts are shown in **Table 2**. There were no significant differences between the two cohorts in baseline parameters. Early death occurred in 17 patients (6.7%) in the training cohort and 9 patients (7.2%) in the validation cohort.

**Table 2:** Characteristics of VSAA Patients at baseline.

Characteristics	Total (n=377)	Training cohort (n=252)	Validation cohort (n=125)	*P* value
**Median age at diagnosis, years (range)**	26(5-75)	27.5(5-75)	25(6-70)	0.312
**Gender, male/female**	203/174	138/114	65/60	0.613
**Median time from diagnosis to IST, days (range)**	18(2-110)	17(2-72)	19(3-110)	0.242
**History, months (range)**	0.75(0.1-132)	1(0.1-108)	0.75(0.17-132)	0.077
**Median ANC, 10^9^/L (range)**	0.05(0-0.19)	0.05(0-0.19)	0.05(0-0.19)	0.965
**Median HGB, g/L (range)**	60(25-123)	59(33-121)	60(25-123)	0.085
**Median PLT, 10^9^/L (range)**	6(0-27)	6(0-27)	6(1-20)	0.786
**Median ARC, 10^9^/L (range)**	3.4(0-38.9)	3.3(0.5-38.9)	3.7(0-37.9)	0.132
**Median SF, ng/mL (range)**	522.45(48.56-3459)	575.1(48.56-3459)	462(64.3-3405)	0.115
**PNH clone (positive/negative)**	66/311	44/208	22/103	0.973
**Fever times before IST, times (range)**	1(0-3)	1(0-3)	1(0-3)	0.560
**Median fever duration before IST, days (range)**	1(0-38)	1(0-38)	1(0-33)	0.808
**Hemorrhagic events before IST, n**	7	5	2	1.000
**Early death, n (%)**	26(6.9%)	17(6.8%)	9(7.2%)	0.870
**Follow-up, months, median (range)**	57(0-137.5)	57(0.1-137.5)	56(0.13-132.1)	0.885

IST, Immunosuppressive therapy; ANC, Absolute neutrophil count; HGB, Hemoglobin; PLT, Platelet; ARC, Absolute reticulocyte count; SF, Serum Ferritin.

### 3.4 Early death risk score model development

According to the ROC curves, we determined the optimal cut-off value was 24 years old for age, 0.015×10^9^/L for the ANC, 900ng/mL for serum ferritin (SF) and 1 time for fever times before IST (**Supplementary Table 2**). Univariate analysis was performed by logistic regression to screen out variables associated with early death of VSAA patients in the training cohort (**Supplementary Table 3**). We checked the variable inflation factors, which ranged from 1.0-1.2, indicating no interactions among these variables in the training cohort. Variables with P <0.1 in the univariate analysis were included in the multivariate analysis to identify independent risk variables for early death. Regression coefficients were rounded to the nearest integer to derive weights to develop the model (**Table 3**). We assigned different points to the variables: 0 point for age ≤24 years old, ANC >0.015×10^9^/L, SF ≤900ng/mL, and fever times before IST ≤1 time; 2 points for fever times before IST >1 time; 3 points for age >24 years old and SF >900ng/mL; 4 points for ANC ≤0.015×10^9^/L (**Supplementary Table 4**).

**Table 3:** Multivariate analysis result of early death in the training cohort.

Variables	Regression coefficient	HR (95%CI)	*P* value	Weighted score
**Age>24 years old**	1.396	4.038 (1.034-15.771)	0.045	3
**ANC ≤ 0.015×10^9^/L**	2.062	7.863 (2.416-25.596)	0.001	4
**SF>900ng/mL**	1.494	4.454 (1.428-13.897)	0.010	3
**Fever times before IST>1 time**	1.163	3.200 (0.953-10.746)	0.060	2

**Figure 3:**
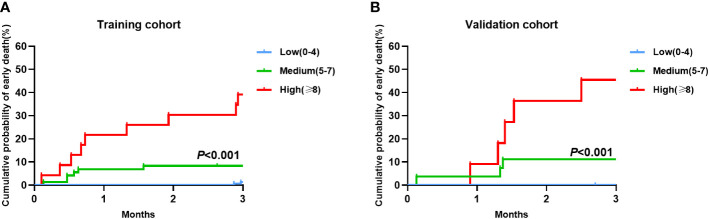
Cumulative probability of early death in the training and validation cohorts by the Early death risk model. **(A)** Training cohort (n=252); **(B)** Validation cohort (n=125).

Early death risk scores were assigned to patients in the training cohort based on different weighted scores, and early death and cumulative early death rates were calculated corresponding to the different scores. We identified three risk levels in the training cohort: low (score 0-4), medium (score 5-7) and high (score ≥ 8) risk (**Table 4**). The early death rate was significantly higher in the medium (8.3%,6/72) compared to the low (1.3%, 2/157) risk group. Early death rate in the high risk group was as high as 39.1% (9/23), much higher than in the medium and low risk groups. In the validation cohort, there were significant differences in the early death rate among the low (1.2%, 1/86), medium (10.7%,3/28), and high (45.5%, 5/11) risk groups as well as the cumulative probability of early death. These results are consistent with the training cohort results (**Figures 3A**, **3B**).

**Table 4:** Early death by risk scores in the training and validation cohorts.

Risk score	Training cohort (n=252)			Validation cohort (n=125)		
	Early death (%, n/N)	The cumulative probability of early death, % (95%CI)	*P* value for trend	Early death (%, n/N)	The cumulative probability of early death, % (95%CI)	*P* value for trend
**Low (0-4)**	1.3%, 2/157	1.3% (0-33.574)	<0.001	1.2%, 1/86	1.19% (0-53.572)	0.001
**Medium (5-7)**	8.3%, 6/72	8.3% (0.094-29.925)	0.019	10.7%, 3/28	11.11% (0.178-41.024)	0.028
**High (≥8)**	39.1%, 9/23	39.1% (16.186-57.535)	<0.001	45.5%, 5/11	45.5% (14.706-72.341)	<0.001
**Overall *P* value**			<0.001			<0.001

### 3.5 Performance of the early death risk score model

**Figure 4:**
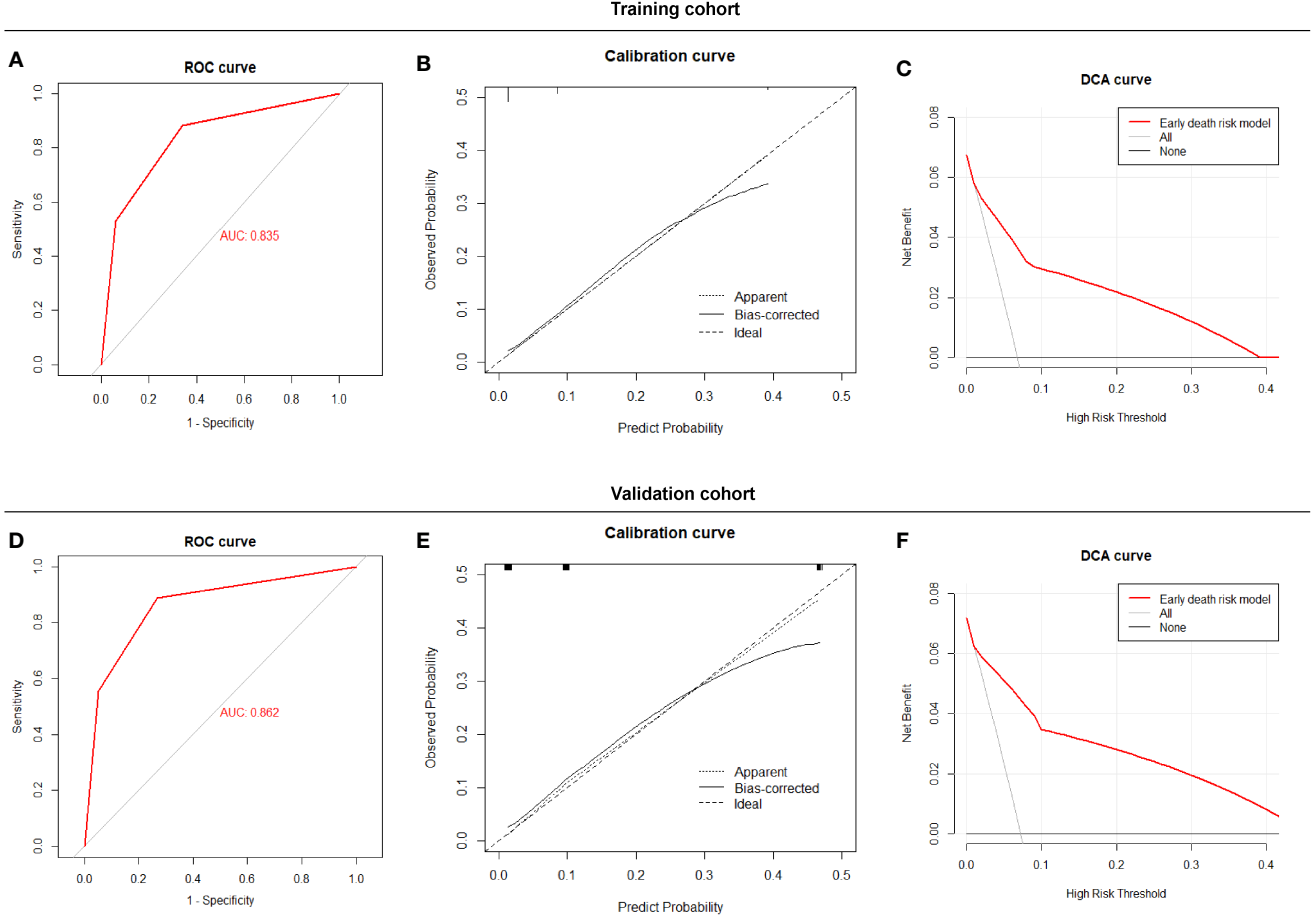
Performance of the Early Death Risk Score model, **(A)** ROC curves of the predictive model for early death in the training cohort **(B)** Calibration plots of the predictive model for early death in the training cohort **(C)** Decision curve analysis of the predictive model early death in the training cohort **(D)** ROC curves of the predictive model for early death in the validation cohort. **(E)** Calibration plots of the predictive model for early death in the validation cohort. **(F)** Decision curve analysis of the predictive model early death in validation cohort.

The Early Death Risk Score model showed good sensitivity and specificity with an AUC of 0.835 (0.734, 0.936) for the training cohort and 0.862 (0.730,0.994) for the validation cohort (**Figures 4A, D**). Calibration plots for probabilities indicated good concordance between predicted and observed outcomes in both cohorts (**Figures 4B, E**). Decision curve analysis indicated good net benefit for clinical application of the model (**Figures 4C, F**).

## 4 Discussion

Currently, treatment guidelines for patients with SAA and VSAA focus on the availability of HLA-matched sibling donors and patient age. However, guidelines do not provide individualized treatment strategies based on disease severity, resulting in some “emergency” patients not receiving what could have been relatively effective treatment. Therefore, this study developed and validated an early death risk score model for VSAA based on baseline characteristics. This risk score model can effectively predict a patient’s early death risk and thereby identify emergency patients, allowing physicians to make better treatment decisions.

The 5-year and 10-year OS was rates were 76.3% and 70.0%, respectively, for the 377 VSAA patients in this study. Another study by the European Society for Blood and Marrow Transplantation (EBMT) (11) found the 10-year OS in VSAA patients treated with IST to be 73%, which is similar to our results. A Chinese study (12) also found that SAA and VSAA patients who underwent HSCT versus those who did not have comparable 6-year OS rates at 75.5% and 76.3%, respectively. Hu et al (13) found that patients with AA undergoing first-line IST had an early-death rate of 7.6%, with 86% of the early death occurring in those with VSAA. In our study, early death occurred in 6.9% of VSAA patients treated with IST, with infection being the leading cause (65.4%, 17/26; 9/17 had two or more multisite complicated infections). Our analysis showed that VSAA patients with early death had extremely low baseline ANC and poor response to G-CSF therapy, and most had multiple, prolonged, complicated infections. Namely, early death was the highest emergency status of VSAA patients.

Therefore, it is vital to construct an early death risk score model for VSAA to identify emergency patients as early as possible for precise medical care of disease. Our results showed that age >24 years old was an independent risk factor for early death in patients with VSAA. Previous studies showed that the effect of age on survival was more significant in patients with VSAA (11, 14, 15). Multivariate analysis revealed that ANC **≤** 0.015×10^9^/L is an independent risk factor for early death, reflecting that extreme depletion of hematopoietic stem or progenitor cells remains an important factor. Similarly, Yagasaki et al. (16) found a poorer IST response in patients with fulminant AA (ANC = 0 for at least 2 weeks prior to and after IST), and more than half (55%, 11/20) of those who failed IST achieved long-term survival only after second-line alternative donor HSCT. Fever times before IST > 1 time indicate severe/recurrent infections in VSAA. Although infection unfavorably affects the prognosis of allogeneic HSCT (4), Xu et al. (17) reported 65 cases of SAA with infection that were successfully treated with HSCT. Recently Liu et al. (18) showed that the 5-year OS of patients with AA experiencing infections was 78.9%, which was similar to the 5-year OS of 81.7% in AA patients without infection. Serum ferritin is an inflammatory biomarker, and SF > 900ng/ml may also reflect infection. However, elevated SF is also associated with iron overload, which can be further identified using other iron metabolism and inflammatory index parameters. In addition, more studies showed that disease severity did not affect post-transplant survival and outcomes in AA (11, 19). In VSAA, early death occurred due to severe and long-term neutropenia. But hematopoietic recovery after IST treatment often took at least 3 months (20), during which the patients were at risk for early death and loss of the opportunity for salvage therapy. In contrast, HSCT can restore neutrophils earlier and provide patients with a better chance of survival (12).

However, we do not believe that all patients with VSAA with a single risk factor for early death should be treated with first line HSCT. Li et al. (21) showed that 119 SAA patients treated with haploidentical donor HSCT had a 3-year OS of 75%. A comparative study by Zhang et al. (22) also found that the 5-year OS in AA cases treated with haploidentical donor HSCT (32% of which was VSAA) and IST (48% of which was VSAA) were 72% (95% CI, 64-84) and 79% (95% CI, 76-89), respectively. This 5-year OS of the haploidentical donor HSCT group was comparable to the 5-year OS of 71.8% for the patients with VSAA in our study who did not experience early death. The early death rate was not high in the low or medium risk groups, whose long-term survival was comparable to that of alternative donor HSCT. In the absence of HLA matching, there is no additional survival benefit even if an alternative donor is chosen as first line. Therefore, HSCT should still be preferred in the presence of an HLA-matched sibling donor for patients with VSAA, consistent with the guidelines (2). And IST should be preferred in the absence of an HLA-compatible sibling donor, with salvage HSCT can be considered when IST is ineffective.

The early death rate of patients with VSAA treated with IST in the high risk group was 39.1%. These patients do not have the opportunity for salvage treatment and should be treated with HSCT as early as possible to increase their chance of survival possibility. A multicenter study by Huang et al (23). found that matched sibling donor HSCT and IST resulted in similar OS in SAA, but in VSAA, the matched sibling donor HSCT group had significantly better OS than IST group (100% vs 85.7%), showing that early transplantation improved patient EFS and hematopoietic reconstitution, providing more benefit. In this study, all patients received the necessary blood product transfusions and G-CSF treatment, and IST was performed in an air laminar flow sterile ward. These interventions well reduced the impact of supportive care issues on early death rate. In recent years, the application of various TPO-RAs, represented by eltrombopag, has led to a new era of AA drug therapy (24–27). Several clinical studies have shown that IST combined with eltrombopag leads to an earlier and better quality of hematologic response, with more prominent effects in SAA (28, 29). In addition, results from a prospective clinical study from EBMT showed that eltrombopag combined with IST as a first-line treatment for AA had a significantly shorter median onset of action than IST alone, but that the median time to first hematologic response was still 3 months (29). Therefore, short term hematologic responses are difficult to obtain in high risk VSAA cases even with TPO-RAs, and early death may occur before IST takes effect. Therefore, HSCT, either with an HLA-matched sibling or an alternative donor, is the only option to increase survival for these patients.

Previous studies focused more on predicting efficacy and survival of IST in patients with AA (4, 30–32). In fact, early identification of patients at risk of early death is even more important. A study by Atta et al (33). that included 185 patients with AA undergoing first-line IST found an early death rate of 15.1% at 3 months after IST. In addition, this study found that age >35 years old and ANC ≤0.1×10^9^/L were independent risk factors for early death and suggested the need for new treatment strategies. Similarly, a study from China (13) included 542 patients with AA undergoing first-line IST (43.5% of which was VSAA) and showed that age >35 years old and insufficient granulocyte reserve were risk factors for early death. These findings are similar to our results. However, our study incorporated multiple covariates and established an early death risk score model, with improved predictive accuracy and that allowed direct assessment of early death risk to provide more clinical benefit.

Our study innovatively proposed an early death risk score model and a refined treatment strategy for VSAA patients. There were several limitations of the study. It was conducted at a single center, was retrospective, and only included patients treated with porcine ATG. While Chinese patients only have access to rabbit and porcine ATG. The porcine form is more widely used and promoted due to its good efficacy, safety, and low price (34–36). Recent articles (36–39) have reported that the overall response and survival rates of AA patients using standard IST with porcine ATG plus CsA are neither better nor worse than those with rabbit ATG. Larger, multicenter studies should incorporate different forms of ATG with and without application use of TPO-RAs for further validation and refinement of the model.

In conclusion, we explored and developed an early death risk score model to identify emergency VSAA patients and guide clinical treatment strategies. Emergency VSAA status is associated with a high risk of early death, and alternative donor HSCT may be a better treatment for these patients than IST, even without HLA matching.

## Data availability statement

The original contributions presented in the study are included in the article/**Supplementary Materials**. Further inquiries can be directed to the corresponding authors.

## Ethics statement

Ethical review and approval was not required for the study on human participants in accordance with the local legislation and institutional requirements. Written informed consent to participate in this study was provided by the participants’ legal guardian/next of kin. Written informed consent was obtained from the individual(s), and minor(s)’ legal guardian/next of kin, for the publication of any potentially identifiable images or data included in this article.

## Author contributions

All authors contributed to the article and approved the submitted version. XL collected data, performed statistical analyses and wrote the manuscript. WY collected and analyzed data, and revised the manuscript. LZ, LJ, LY, KZ, YL, JL HF, YY, and YX collected data and followed up with patients. XZ and FZ designed the study, supervised the data analysis, and revised the manuscript. All authors contributed to the article and approved the submitted version.
