# Supplementary material for: Development and validation of early death risk score model for emergency status prediction in very severe aplastic anemia
Source: Front Immunol. 2023 Apr 20;14:1175048. doi: 10.3389/fimmu.2023.1175048 (PMC10158980; doi:10.3389/fimmu.2023.1175048)
Supplement: Supplementary file 1 [file Table_1.docx]

Supplementary Material

Development and validation of Early Death Risk Score Model for emergency status prediction in very severe aplastic anemia

**Xu liu^1,2†^, Wenrui Yang^1,2†^, Li Zhang^1,2^, Liping Jing^1,2^, Lei Ye^1,2^, Kang Zhou^1,2^, Yuan Li^1,2^, Jianping Li^1,2^, Huihui Fan^1,2^, Yang Yang^1,2^, Youzhen Xiong^1,2^, Xin Zhao^1,2*^, Fengkui Zhang^1,2*^**

*** Correspondence:** Xin Zhao^*^; Email: [zhaoxin@ihcams.ac.cn](mailto:zhaoxin@ihcams.ac.cn)

Fengkui Zhang^*^; Email: [fkzhang@ihcams.ac.cn](mailto:fkzhang@ihcams.ac.cn)

# Supplementary Data

| **Supplementary Table 1. Hematologic Response of Non-early death patients (n=351).** | | |
| --- | --- | --- |
| **Hematological Response** | **Response at 3m, n (%)** | **Response at 6m, n (%)** |
| **OR(CR+PR)** | 93 (26.5%) | 147 (41.9%) |
| **CR** | 14 (4.0%) | 37 (10.5%) |
| **PR** | 79 (22.5%) | 110 (31.3%) |
| **NR** | 258 (73.5%) | 204 (58.1%) |

| **Supplementary Table 2. Optimal cut-off values for baseline parameters by ROC analyses.** | | | | | | |
| --- | --- | --- | --- | --- | --- | --- |
| **Parameters** | **AUC** | **95%CI** | **Sensitivity (%)** | **Specificity (%)** | **Youden index** | **Cut-off value** |
| **Age, years** | 0.677 | 0.572-0.782 | 84.6 | 44.7 | 0.293 | 23.5 |
| **Time from diagnosis to IST, days** | 0.428 | 0.291-0.566 | 19.2 | 90.6 | 0.098 | 28.5 |
| **History, months** | 0.558 | 0.442-0.674 | 88.5 | 23.1 | 0.115 | 0.49 |
| **ANC, 10^9^/L** | 0.784 | 0.684-0.883 | 79.8 | 65.4 | 0.452 | 0.015 |
| **HGB, g/L** | 0.464 | 0.367-0.560 | 100 | 12.8 | 0.128 | 47.5 |
| **PLT, 10^9^/L** | 0.406 | 0.296-0.516 | 3.8 | 99.1 | 0.030 | 19.5 |
| **ARC, 10^9^/L** | 0.744 | 0.662-0.825 | 70.4 | 69.2 | 0.396 | 2.55 |
| **SF, ng/mL** | 0.661 | 0.540-0.783 | 57.7 | 76.6 | 0.343 | 915.45 |
| **Fever times before IST, times** | 0.714 | 0.593-0.835 | 88.2 | 43.8 | 0.321 | 0.5 |
| **Fever duration before IST, days** | 0.701 | 0.595-0.807 | 50 | 82.3 | 0.323 | 5.5 |

| **Supplementary Table 3. Univariate analysis result of early death in the training cohort.** | | | |
| --- | --- | --- | --- |
| **Variables** | **Regression coefficient** | **HR (95%CI)** | ***P* value** |
| **Gender** | -0.177 | 0.837 (0.308-2.275) | 0.728 |
| **Age > 24 years** | 1.378 | 3.969 (1.111-14.175) | 0.034 |
| **Age > 40 years** | 0.605 | 1.831 (0.669-5.013) | 0.239 |
| **ANC ≤ 0.015×10^9^/L** | 2.184 | 8.880 (2.988-26.386) | 0.000 |
| **PLT ≤ 5×10^9^/L** | 0.700 | 2.013 (0.721-5.623) | 0.182 |
| **HGB ≤ 70 g/L** | 18.703 | 132671823.2(0-) | 0.998 |
| **ARC ≤ 3×10^9^/L** | 1.911 | 6.757 (1.890-24.152) | 0.003 |
| **SF > 900ng/mL** | 1.421 | 4.143 (1.510-11.369) | 0.006 |
| **Fever before IST** | 1.767 | 5.852 (1.309-26.169) | 0.021 |
| **Fever times before IST > 1 time** | 1.455 | 4.285 (1.524-12.046) | 0.006 |
| **Fever duration before IST >5 days** | 1.914 | 6.783 (2.391-19.242) | 0.000 |
| **No response to G-CSF** | 18.894 | 160602742.9 (0-) | 0.997 |
| **Hemorrhagic event before IST** | 2.333 | 10.311 (1.599-66.490) | 0.014 |

| **Supplementary Table 4. Calculation of the early death risk scores.** | | | |  |
| --- | --- | --- | --- | --- |
| **Variables** | **Risk Points** | | | |
|  | **0** | **2** | **3** | **4** |
| **Age > 24 years** | No | - | Yes | - |
| **ANC ≤ 0.015×10^9^/L** | No | - | - | Yes |
| **SF > 900ng/mL** | No | - | Yes | - |
| **Fever times before IST > 1** | No | Yes | - | - |
